# Editorial: Systematic reviews of pharmacological and non-pharmacological psychiatric interventions

**DOI:** 10.3389/fpsyt.2023.1201888

**Published:** 2023-04-25

**Authors:** Mirko Manchia, Joe Kossowsky

**Affiliations:** ^1^Section of Psychiatry, Department of Medical Sciences and Public Health, University of Cagliari, Cagliari, Italy; ^2^Unit of Clinical Psychiatry, University Hospital Agency of Cagliari, Cagliari, Italy; ^3^Department of Pharmacology, Dalhousie University, Halifax, NS, Canada; ^4^Department of Anesthesiology, Critical Care and Pain Medicine, Boston Children's Hospital, Harvard Medical School, Boston, MA, United States; ^5^Department of Anaesthesia, Harvard Medical School, Boston, MA, United States

**Keywords:** meta-analysis, data synthesis, clinical trials, intervention, severe mental disorders, biostatistics

Systematic reviews aim to search, appraise, and synthesize all relevant empirical evidence related to a specific research question in an unbiased and well-documented fashion. Ideally, they provide readers with a complete interpretation of research results and enable them to critically appraise the literature (1). Given the fast pace of medical research, systematic reviews are becoming increasingly important in providing clear, comprehensive, and reproducible overviews of available evidence and identifying research gaps in the field. Psychiatry has not been an exception in this regard, particularly considering the relatively high rates of patients experiencing inadequate response to treatments (2). Indeed, suboptimal or non-response can manifest during pharmacological as well as non-pharmacological treatments. In this regard, it is of interest that the first conceptualization of the term resistance was developed in the context of psychoanalysis (3). Diverse strategies, such as for instance augmentation, combination therapies, and intensive clinical monitoring can influence substantially treatment outcomes. Thus, qualitative and quantitative data synthesis might guide treatment decision making and effective personalized management of patients affected by mental disorders. In this context, the goal of our Research Topic was to assemble systematic reviews (and when appropriate, meta-analyses) of Pharmacological and Non-Pharmacological Interventions for psychiatric disorders and/or in psychiatric and special populations. The results of these systematic reviews and meta-analyses offered insights on diverse interventions stemming from complementary and alternative medicine to neurocognitive training and behavioral approaches, and from biological treatments to physical exercise. In addition, several relevant outcomes were the object of the studies collected in this Research Topic, including drug adverse effects, violent behavior, agitation, depression, and neurocognitive impairment.

Concerning complementary and alternative treatments, in a systematic review and meta-analysis of 30 RCTS including over 5,000 patients, Rao et al. investigated the efficacy and safety of Traditional Chinese herbal medicine compared to Western Medicine in treating antipsychotic-related constipation. Results indicated that Traditional Chinese herbal medicine was associated with significantly better moderate and marked response rates, as well as remission rates, as compared to Western Medicine. In addition, the adverse effect of rash was significantly less frequent in Traditional Chinese herbal medicine than in Western Medicine. Although authors noted that more high-quality studies are necessary for generalizing findings, results still highlight the safety and efficacy of Traditional Chinese herbal medicine as a treatment for antipsychotic-related constipation in clinical practice. Furthermore, in a systematic review and meta-analysis of 25 studies and 2,213 women, Zhao et al. examined the efficacy of acupuncture for the treatment of perimenopausal depression. Specifically, this study investigated the efficacy of acupuncture independently, and in conjunction with standard care (i.e., antidepressant/hormone replacement therapy), as compared to waitlist control or placebo/sham acupuncture (Zhao et al.). Primary findings suggested that acupuncture outperformed standard care in reducing depressive symptoms, though when used in conjunction with standard care, acupuncture as an adjuvant treatment was more effective in reducing depressive symptoms as compared to either acupuncture alone or standard care alone. The comparative efficacy between acupuncture and placebo/sham acupuncture, however, could not be determined due to a lack of RCTs on this Research Topic. Still, findings indicate the clinical utility of acupuncture as an independent and adjuvant treatment alongside standard care for perimenopausal depression.

Another set of articles examined the efficacy of non-pharmacological interventions in disruptive behaviors such as aggression and violence, neurocognitive impairment and eating disorders. Slamanig et al. conducted a systematic review of 10 non-pharmacological interventions aimed at reducing risk of violence in individuals with schizophrenia spectrum disorders (SSD) in forensic psychiatry settings. These interventions included neurocognitive training, cognitive-behavioral treatment programs, and other interventions, such as an integrative treatment program, among others (Slamanig et al.). Results revealed significant methodological limitations across included studies including small sample sizes, lack of randomization, and lack of control group comparisons; results also indicated no clear trends of effectiveness for such interventions. As such, the authors did not set forth any firm conclusions about the efficacy of non-pharmacological interventions, emphasizing, instead, the need for sufficiently powered RCTs with more reliable operationalizations of “violence” and diagnostic specificity of SSD individuals. Moreover, Stuchlíková and Klírová conducted a mini-review investigating the effects transcranial direct current stimulation (tDCS), which is a non-invasive, low-current neurostimulation method, on the positive, negative, and cognitive symptoms of schizophrenia. This review included 27 randomized controlled parallel-group design studies with 966 patients, and studies were grouped according to their focus on either positive (10 studies), negative (five studies), or cognitive (12 studies) symptoms of schizophrenia. In general, results revealed that tDCS demonstrated efficacy in each symptom domain (Stuchlíková and Klírová). More specifically, six of 10 studies assessing positive symptoms showed improved outcomes, all studies assessing negative symptoms showed improved outcomes, and eight or 12 studies assessing cognitive outcomes showed improved outcomes. Notably, tDCS also emerged as a well-tolerated and safe method of intervention in reviewed studies. The authors concluded that tDCS has clinical promise for addressing wide-ranging symptomatology of schizophrenia. In a systematic review of individuals in forensic settings with acquired brain injuries (ABI), de Geus et al. investigated the efficacy of non-pharmacological interventions, particularly those aimed at supporting the cognitive, emotional, and behavioral changes associated with ABI. In sum, four studies were examined in this review, including two case design studies and two single group experimental designs, with a total of 86 individuals included across the four studies. Results identified the relative efficacy for non-pharmacological interventions for individuals with ABI, including improvements in cognitive functioning, increases in productivity, and decreases in aggression and recidivism (de Geus et al.). Generalized conclusions about non-pharmacological interventions for ABI from this review, however, are tempered by the lack of methodologically rigorous studies conducted thus far on this Research Topic. Finally, Toutain et al. conducted a systematic review of 27 studies aimed at investigating the efficacy of exercise therapy (ET) for individuals with anorexia nervosa in inpatient and outpatient settings. This review examined specifically the effects of four types of ET, including aerobic exercise, resistance exercise, mind-body physical exercise, and combined physical exercise, on anorexia nervosa symptomatology, physical health outcomes, and mental health outcomes. Overall, results indicated that ET had significant, positive effects on outcomes of interest, though specific associations varied by type of ET. That is, results revealed that aerobic and resistance exercise was associated with increased muscle strength and that mind-body physical exercise was associated with improved eating disorder and mental health symptoms. ET that combined different physical exercises was associated with increased weight gain and reduced dysfunctional exercise. Taken together, these studies suggest that ET may be an effective intervention for improved functioning in individuals with anorexia nervosa (Toutain et al.). Authors emphasize, however, that conclusions should be interpreted with caution due to the lack of well-designed RCTs as well as the insufficient details provided about ET interventions, thus limiting reproducibility of findings.

Another two reviews examined the efficacy of pharmacological treatments in substance intoxication and agitation. In their systematic review of 11 RCTs, Amore et al. examined the use of lorazepam for acute agitation in adult patients with mental or behavioral disorders. Overall, findings suggested that lorazepam is an effective treatment of agitation, though some more nuanced results emerged. Specifically, in five studies, the combination of haloperidol and lorazepam was more effective than either medication alone, though lorazepam was not significantly more effective than haloperidol, individually. In another study, olanzapine was more effective than lorazepam. In general, the most frequent side effects that emerged alongside lorazepam treatment were dizziness, sedation, and somnolence, suggesting its relative safety for clinical use. Moreover, in a review and analysis of 51 case reports, Ordak et al. investigated pharmacotherapy responses for patients presenting with effects of new psychoactive substances. Results revealed that most patients had ingestion of synthetic cathinones or cannabinoids. In terms of pharmacotherapy, most patients (62.7%) were administered benzodiazepines in response to the effects of taking new psychoactive substances, and these were primarily prescribed to reduce patient psychomotor agitation and aggression. In general, the number of medications prescribed to patients increased over time (i.e., length of hospitalization; Ordak et al.). Of note, five case reports indicated a patient fatality, with the majority of these patient deaths due to patient ingestion of synthetic opioids. Taken together, these findings highlight the overall importance of safe management of psychomotor agitation with benzodiazepines for patients taking new psychoactive substances.

The last study by Lin et al. applied software for scientometric visual analysis, investigating trends in research on the topic of GABAergic networks in depression in recent years (i.e., 2004–2020). Results revealed that research in this area has increased significantly overtime, as measured by increasing numbers of publications on GABAergic networks in depression, particularly in the past 5 years (Lin et al.). With respect to clinical and research implications of results from this review, findings highlight the importance of the development of pharmacological interventions that enhance the transmission of GABA for most effective treatment of depression.

In summary, this Research Topic showed that data synthesis is a key component of psychiatric research especially in those areas where treatment options remain suboptimal, engulfed by safety issue, and/or limited by the absence or properly designed trials. The identification of novel treatments through these analytical approaches might promote new lines of clinical and basic research as well as guide clinicians in choosing concomitant treatment options that may increase the rate of response in treated patients.

## Author contributions

All authors took part in drafting the article, revising it critically for important intellectual content, gave final approval of the version to be published, and agree to be accountable for all aspects of the work.

## References

[1] Aromataris E Pearson A. The systematic review: An overview. Am J Nurs. (2014) 114:53–8. 10.1097/01.NAJ.0000444496.24228.2c 24572533

[2] Howes OD Thase ME Pillinger T. Treatment resistance in psychiatry: State of the art and new directions. Mol Psychiatry. (2021) 27:58–72. 10.1038/s41380-021-01200-3 34257409 PMC8960394

[3] Sigmund F. A general introduction to psychoanalysis. J Nerv Mental Dis. (1920) 52:548–9.

